# One-Year Outcomes Using Ranibizumab for Neovascular Age-Related Macular Degeneration: Results of a Prospective and Retrospective Observational Multicentre Study

**DOI:** 10.1155/2011/405724

**Published:** 2011-11-28

**Authors:** Lars Hjelmqvist, Charlotte Lindberg, Pär Kanulf, Henrik Dahlgren, Ingrid Johansson, Annica Siewert

**Affiliations:** ^1^St. Erik's Eye Hospital, Karolinska Institutet, Polhemsgatan 50, 11282 Stockholm, Sweden; ^2^Department of Ophthalmology, Lund University Hospital, 22185 Lund, Sweden; ^3^Department of Ophthalmology, Helsingborg Hospital, 25187 Helsingborg, Sweden; ^4^Department of Ophthalmology, Ryhov Hospital, 55185 Jönköping, Sweden; ^5^Department of Ophthalmology, Södersjukhuset, 11883 Stockholm, Sweden; ^6^Department of Ophthalmology, Örebro University Hospital, 70185 Örebro, Sweden; ^7^Medical Department, Novartis Sweden AB, 18311 Täby, Sweden

## Abstract

The Swedish Lucentis Quality Registry is a 12-month, open-label, observational, prospective, and retrospective study of ranibizumab administration for wet AMD. Visual acuity (VA) was measured with Snellen or ETDRS chart in 370 patients (66.8% women; age range 46–93 years). In total, a mean of 4.7 ± 1.6 injections per patient (range 1–10) was given to month 12. Mean VA score was 58.3 ± 12.2 letters before treatment, 63.3 ± 12.5 after 3 injections (Δ4.9 ± 10.1 letters from baseline), and 59.3 ± 16.2 at 12 months (Δ1.0 ± 13.6). VA score from baseline to month 12 was stable in 74.4% of patients, improved by 15 letters/3 lines or more in 14.7%, and decreased by ≥15 letters/3 lines in 10.9% of patients. With a mean of 4.7 ranibizumab injections per patient per year, mean VA was stabilised but not increased. To maintain the initial gain seen after the first three injections, an average of 1.8 ± 1.5 additional injections does not appear to be adequate.

## 1. Introduction

As the population ages, establishing effective treatment strategies for age-related macular degeneration (AMD) becomes increasingly important. AMD is the leading cause of irreversible blindness in patients over the age of 50 [1–3], with an incidence that rises from 0.2% in those aged 55–64 to 13% after the age of 85 [4]. The neovascular form of AMD, characterised by choroidal neovascularisation and proliferation of fibrous tissue, represents only 10–15% of cases but is responsible for more than 80% of AMD-related severe visual loss or blindness [5].

Management of neovascular AMD centres on intravitreal antiangiogenic therapy, which localises therapy to the eye and avoids systemic exposure. Following limited success with pegaptanib, the first licensed intravitreal agent for neovascular AMD [6], ranibizumab, became available in 2006. Ranibizumab is an antivascular endothelial growth factor (VEGF) antibody fragment which binds to VEGF and inhibits the contribution of VEGF to the formation of neovascular lesions in the choroid [7]. Two pivotal trials in which ranibizumab was injected intravitreally once a month, one placebo-controlled and the other using Photodynamic Therapy (PDT) as the control, both showed a remarkable improvement in visual acuity (VA) from baseline in treated eyes, as measured by the Early Treatment Diabetic Retinopathy Study (ETDRS) chart [8, 9]. On the basis of these results, ranibizumab is now recommended as a first-line therapy [10] and is the most widely prescribed treatment for neovascular AMD.

Nevertheless, there remain unanswered questions about the optimal evaluation and treatment regimen for ranibizumab that balances VA improvement in the individual patient versus logistical and cost issues. Recommendations for the use of ranibizumab have been developed which point out that continued monthly injections offer the best VA outcomes but that if regular monthly administration is not feasible then flexible retreatment after three monthly injections is viable [11]. In practice, however, the current consensus is that after the first three injections, monthly maintenance visits with clinician-determined retreatment are appropriate [10, 12]. Evidence concerning the efficacy of administering ranibizumab as needed based on monthly evaluation by clinical findings or imaging is growing, with two small, nonrandomised prospective studies [13, 14], a series of retrospective analyses [15–21] and most recently a large multicentre, single-blind trial [22].

As part of an ongoing pharmacovigilance program for ranibizumab, Novartis Pharma AG (Basel, Switzerland) has initiated the LUMINOUS program designed to assess long-term safety, efficacy, treatment patterns, and health-related quality of life outcomes in a large number of patients treated with ranibizumab in routine clinical practice across the world. We report here the findings from the Swedish Lucentis Quality Registry, which records efficacy and safety results following ranibizumab administration according to local practice in patients with AMD at five specialist centres. The aim of the registry is to understand how ranibizumab is being deployed in routine practice outside the setting of a clinical trial, and to establish what improvement in VA can be achieved and the number of injections and visits needed. The current paper addresses the following objectives of the registry: (i) to characterise the population of patients receiving ranibizumab, (ii) to record the number of injections administered and the dosing frequency, (iii) to evaluate the effect of ranibizumab treatment on VA, and (iv) to characterise, describe, and evaluate side effects. Data collected up to January 2011 are presented.

## 2. Methods

### 2.1. Study Design

The Swedish Lucentis Quality Registry is a 12-month, open-label, observational, noncomparative study that is ongoing at five specialist centres in Sweden. These clinics were chosen since they were among the first to implement intravitreal ranibizumab treatment for wet AMD in Sweden. Outpatients who were receiving ranibizumab at the time the study started (retrospective component) or with whom the decision was subsequently made to start treatment with ranibizumab (prospective component) were eligible for inclusion unless they were receiving the drug within a controlled clinical trial. Ranibizumab treatment is administered according to the approved label, that is, three initial injections and then according to need based on VA, optical coherence tomography (OCT) findings, and the investigator's judgment. The assessments performed and the criteria applied at each centre to initiate treatment or reinjection were not recorded. Standard single-use ranibizumab vials (containing 0.23 mL ranibizumab 10 mg/mL) are used.

Retrospective data were collected from patients who started ranibizumab during the period from July 2007 to March 2008. The prospective component was initiated in April 2008, when the registry was opened, with recruitment ending in December 2009. Written, informed consent was obtained from all participants following ethical approval from the Regional Ethical Review Board in Stockholm.

### 2.2. Evaluation and Data Collection

All clinical assessments are performed at the discretion of the investigator as per local practice. Data are recorded at every monthly visit during the first 12 months of treatment. For both retrospectively and prospectively included patients, the visits that took place closest to these times were used for evaluation. Retrospective data were obtained from patients' medical records. Prospective data are collected at study entry (i.e., the date of first ranibizumab injection) and during the following year, in accordance with the local visit schedule.

The following data are obtained at baseline and at all routine visits during the first year of ranibizumab treatment: patient age and gender (baseline only); indication for use of ranibizumab; cause of treatment discontinuation prior to the end of the 12-month observation, if applicable; number, timing, and frequency of ranibizumab doses administered during the first 12 months of treatment; whether OCT was or was not performed at baseline; VA prior to treatment and at all visits (except at the second and third injection visits when VA was not always measured) during the first 12 months of treatment as assessed by the ETDRS chart and adverse events since study entry. In addition, vision-related function as measured by The National Eye Institute Visual Function Questionnaire-25 (VFQ-25) is recorded at study entry, 3 months and 12 months in the prospectively recruited patients. VFQ-25 subscales were calculated as per the recommended procedure [23]. Each item was converted to a 0-to-100 scale where higher scores represent better functioning. Items within a subscale were averaged and the score represents the average for all items in the subscale. The overall score was averaged over all subscales, excluding the general health item, and thus equal weight was given to each subscale.

All data are entered online by the clinical team at each centre to a password-protected web-based data system. Access to individual patient data is only available to the local clinical team. All participating centres have online access to pooled data in real time from their own centre and from the total patient population.

### 2.3. Data Analysis

Assessment of efficacy is based on VA measurements (ETDRS chart scores) before and after treatment. In 100 of the retrospective patients, Snellen VA testing was used at baseline. In order to use these data, the Snellen value was recalculated to determine the corresponding ETDRS value using a formula that reflects the relationship between the two methods:

(1) $$\text{ETDRS}\;\left(\text{number}\;\text{of}\;\text{letters}\right)=50^{*}\log_{10}\left(\frac{\text{Snellen}}{100}\right)+36.$$

VA measurements are presented as absolute values (mean ± standard deviation [SD]), as a mean change from baseline to month 12 (mean ± standard error [SEM]), and in terms of the number of patients with (i) a gain of ≥15 letters or 3 lines, (ii) a change of <15 letters or 3 lines, or (iii) a loss of ≥15 letters or 3 lines.

The VFQ-25 subscales are not strictly ordinal or equal interval measures, but because they approximate interval-level measures, mean and standard deviations were computed [24]. Change from baseline to 3 and 12 months was tested with Wilcoxon signed rank test. Since the probability of making type 1 error increases with the number of analytical tests performed, care should be taken when interpreting the results.

Safety assessment was based on the frequency of adverse events and serious adverse events. Adverse events were categorised by severity (mild, moderate, or severe), relationship to ranibizumab (probable, possible, or unlikely), and whether they constituted a serious adverse event.

Data are presented for the on-treatment population, comprising all intent-to-treat (ITT) patients in whom treatment/followup visits were not discontinued during the one-year study period. All data are presented descriptively.

It was anticipated that retrospective data from approximately 200 patients would be available, with prospective data from a further 200 patients. No sample size calculation was required since no statistical hypothesis was tested.

The study database and electronic data capture were managed by Pharma Consulting Group AB, Uppsala, Sweden. Data analysis was undertaken by the Pharma Consulting Group using the SAS system version 9.2 (SAS Institute Inc, Cary, NC, USA).

## 3. Results

### 3.1. Patient Population

In total, 475 patients were enrolled in the registry, of whom 471 received at least one dose of ranibizumab and formed the ITT population. Two hundred and seventy-two patients were enrolled retrospectively [57.7%] and 199 prospectively [42.3%]. Of these, 370 patients were followed for one year without discontinuing ranibizumab treatment/followup visits (the “on-treatment” population; retrospective 206 [55.7%], prospective 164 [44.3%]). In total, 101/471 patients (21.4%) discontinued before one year. In approximately a third of these cases (*n* = 34), this was due to the treating physician's decision that no further followup was required (Table 1). The 43 patients who discontinued for “other reasons” did so for a variety of causes, the most frequent being VA was too low (*n* = 17), lack of VA improvement (*n* = 3), nonattendance at followup visits (*n* = 7), change in therapy (*n* = 6), death (*n* = 5), and various reasons (*n* = 5). There were no marked differences between the ITT and on-treatment populations in terms of demographics or baseline characteristics (Table 1).

Two-thirds of the population were female, and the mean age at entry was 78 years (Table 1). Ranibizumab injections were evenly divided between left and right eyes (52.7% and 47.3%, resp.). The mean number of visits during the first year of treatment in the on-treatment population, irrespective of whether ranibizumab was injected, was 9.4 ± 1.8, ranging from 4 to 13 visits. The last visit at which VA was assessed occurred at months 7, 8, 9, 10, 11, 12, and 13 in 1, 6, 22, 27, 50, 262, and 2 patients, respectively.

### 3.2. Ranibizumab Therapy

**Figure 1:**
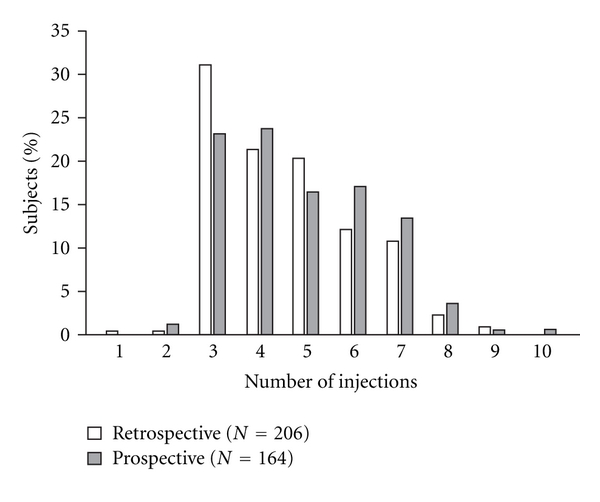
Number of ranibizumab injections received during year 1 in the retrospectively and prospectively recruited subpopulations (on-treatment population, *n* = 370).

All three initial ranibizumab injections were administered to 95.1% (352/370) in the on-treatment population. The mean number of ranibizumab injections was 4.7 ± 1.6 (mean 1.8 ± 1.5 after the three initial injections). The number of injections in the on-treatment population was similar in the retrospective subpopulation (mean 4.6 ± 1.5; 197/206 [95.6%] received all three initial injections) and the prospective subpopulation (mean 4.9 ± 1.6; 155/164 [94.5%]). The majority of patients received three (27.6%, *n* = 102), four (22.4%, *n* = 83), or five (18.6%, *n* = 69) injections, with a slightly higher proportion of prospectively enrolled patients receiving more than three injections than in the earlier, retrospective cohort (Figure 1). There was no correlation between VA at baseline and the number of ranibizumab injections given (data not shown).

### 3.3. Visual Acuity

**Figure 2:**
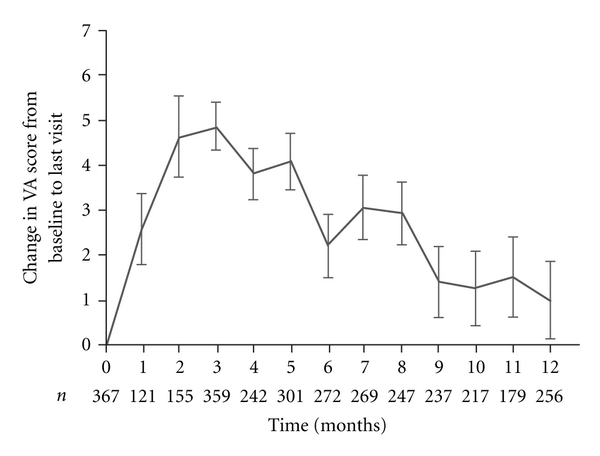
Change in VA score (ETDRS chart) from baseline (BL) to month 12 (on-treatment population, *n* = 370). Values are shown as mean ± SEM.

The mean VA score at baseline was 58.3 ± 12.2 letters (on-treatment population) and was similar in the retrospective and prospective subpopulations (58.2 ± 12.0 letters and 58.4 ± 12.3 letters, resp.). At month 12, the mean VA score was 59.3 ± 16.2 letters, an increase of 1.0 ± 13.6 versus baseline. The maximum improvement was seen at month 3 following administration of three ranibizumab injections, when mean VA score peaked at 63.3 ± 12.5 letters, a mean increase of 4.9 ± 10.1 from baseline. After month 3, the mean VA score progressively declined back to baseline levels (Figure 2). The mean improvement in VA score from baseline to month 12 was greater in younger patients and in females (Table 2). There was no consistent association between improvement in VA score and the number of injections administered. 

The majority of patients (74.4%) showed a stable score over the 12-month period, defined as a change of <15 letters/3 lines. In total, 14.7% (*n* = 54) had improved by 15 letters/3 lines or more, while 10.9% of patients (*n* = 40) had lost ≥15 letters/3 lines (Table 2).

### 3.4. Vision-Related Function (VFQ-25)

The increase in VA at month 3 was accompanied by a significant improvement in VFQ-25 total score from baseline, as recorded in 131 of the prospectively recruited patients (Table 3). Several VFQ-25 subscales, including general vision, ocular pain, and both near and distance activities, also improved significantly. By month 12, the general vision, near activities, and driving subscales were significantly higher compared to baseline, but total VFQ-25 score had returned to near-baseline levels. 

### 3.5. Safety and Tolerability

In the ITT population, that is, all patients who received one or more ranibizumab injection, a total of 17 adverse events occurred in 16 patients during followup (16/471 [3.4%]). Of these, eight were graded mild (pain (2), conjunctivitis (2), retinal pigment epithelial tear, conjunctival haemorrhage, blepharal papilloma, and transient ischaemic attack), four moderate (retinal pigment epithelial tear (2), angina pectoris, and cardiac failure), and four severe (duodenal ulcer haemorrhage, cerebrovascular accident, mesothelioma, and death). The treating physician considered there to be a probable relation to ranibizumab in two cases (mild conjunctival haemorrhage that did not require treatment and retinal pigment epithelial tear in a patient with very low VA before treatment and after one injection the treatment was stopped due to low vision), and a possible relation in five cases: mild pain (2), cerebrovascular accident, transient ischaemic attack, and retinal pigment epithelial tear (following which ranibizumab treatment was temporarily stopped). There were four deaths during the study, none reported as related to the treatment. 

## 4. Discussion

The Swedish Lucentis Quality Registry provides a large database relating to clinical experience with ranibizumab treatment for neovascular AMD, for which almost half the patients were recruited prospectively. In this cohort of 370 patients followed for one year at five centres, almost all patients (95%) received three initial monthly injections as per the product license, with an average of 1.8 subsequent injections. The improvement in VA following the initial three injections (mean 4.9 letters) was somewhat lower than that observed in the pivotal MARINA [8] and ANCHOR [25] trials (~6 and ~10 letters, resp.). With the current treatment pattern, this benefit was not sustained: the VA score at month 12 was similar to baseline. 

However, a higher rate of subsequent ranibizumab injections could be expected to have sustained the initial improvement in VA seen after the first three administrations. VEGF level has been shown to correlate with the extent of macular oedema [26], and while the decrease in VEGF level observed following intravitreal injection of ranibizumab is lost after four weeks it is prolonged by retreatment [26]. In the 24-month MARINA [8] and ANCHOR [9, 25] studies, a fixed monthly schedule of ranibizumab showed sustained benefits to the end of each trial. In an attempt to minimise the number of injections administered, a quarterly ranibizumab treatment regimen has been investigated in randomised studies [27–30] but using this approach the sustained improvement in VA achieved with three monthly injections was lost. An individualised dosing strategy has been explored in a small nonrandomised, open-label trial (PrONTO) [13] which employed three initial monthly injections of ranibizumab with subsequent injections performed based on the evolution of VA and the presence or absence of subfoveal fluid, as detected by OCT. The mean VA improvement described in the PrONTO study was similar to that seen in the MARINA and ANCHOR trials, but required an average of 5.6 injections over the first year [13], with the improvement maintained at two years [31]. Another nonrandomised prospective study, in which patients received a mean of 5.1 clinically determined ranibizumab injections over 12 months, with a single application of reduced fluence photodynamic therapy, reported a mean VA improvement of 7.2 letters at month 12 [14]. Other recent studies have demonstrated a higher response as the number of injections increases [21, 32]. The most conclusive evidence comes from the recent CATT trial, a large randomised study in which ranibizumab given monthly or as needed was compared to bevacizumab, again administered monthly or as needed on the basis of monthly evaluations [22]. Results showed that the efficacy of ranibizumab at one year, as measured by improvement in VA, was similar with either monthly or individualised dosing, with the individualised group receiving a mean of 6.9 injections by month 12. In our observational study, a mean of 4.7 injections resulted in stable VA with no long-term improvement although elsewhere an individualised, clinically driven approach to reinjection has demonstrated good results obtained with a mean of >5 reinjections after the initial three-injection regimen [29, 33, 34]. It appears that the number of reinjections may be critical in maintaining early increase in VA and that fewer than 5 injections during the first year appear inadequate.

No marked difference in the change in VA score was observed between the retrospective and prospective subpopulations (to be confirmed), although slightly more prospectively recruited patients received >3 injections. One hundred of the 272 patients in the retrospective population had baseline VA measured by Snellen scoring, requiring conversion to EDTRS values. The two scoring systems are known to show differences, particularly in patients with wet AMD and poor VA [35], but the similarity of VA score at baseline in the retrospective cohort (including patients converted from Snellen scores) and the prospective arm suggests that this was not an important source of bias.

The change in VA score over the 12-month followup period was mirrored in the observed change in vision-related function. A significant improvement in VFQ-25 total score was achieved at month 3 but declined to near-baseline levels by month 12. Certain subscale scores remained significant at month 12, including general vision and near activities, but the relatively small number of patients (~90) and the subjective nature of scales of this type mean that this should be interpreted with caution when the improvement in the more robust endpoint of VA score was not maintained to month 12.

Regarding safety, intravitreal ranibizumab has previously been established as a safe and well-tolerated therapy for neovascular AMD [36]. In our population of 471 patients, there were only two adverse events with a probable relation to ranibizumab (a mild conjunctival haemorrhage and a pigment epithelium rift) over the 12-month study period, with a possible relation in a further five cases. No patient discontinued treatment due to adverse events. This safety profile is consistent with the extremely low systemic exposure to ranibizumab observed following intravitreal administration and the short half-life of the drug [37, 38], which minimises the risk of systemic anti-VEGF adverse events.

## 5. Conclusion

The findings from this large cohort of retrospectively and prospectively recruited patients indicate that an individualised approach, whereby three monthly initial injections are followed by additional injections as determined by OCT or clinical monitoring, can maintain VA at long-term baseline levels. To sustain the short-term improvement seen after the initial injection schedule, however, requires more than the mean of 1.8 repeat injections administered in this population. The observational nature of the study does not, however, permit specific recommendations on the optimal number or frequency of injections or the indications for reinjection.

## Figures and Tables

**Table 1:** Baseline characteristics and ranibizumab treatment.

	ITT population (*n* = 471)	On-treatment population (*n* = 370)
Baseline age (years)		
Mean ± SD	78.1 ± 8.0	77.7 ± 8.0
Range	46–93	46–93

Gender, *n* (%)		
Male	160 (34.0%)	123 (33.2%)
Female	311 (66.0%)	247 (66.8%)

Indication for ranibizumab, *n* (%)		
Wet AMD	468 (99.4%)	368 (99.5%)
Other	3 (0.6%)	2 (0.5%)

Baseline OCT performed, *n* (%)		
Yes	357 (75%)	242 (75%)
No	115 (24%)	82 (25%)
Unknown	2 (0.4%)	0

Baseline ETDRS, *n* (%)		
Yes	337 (71%)	223 (69%)
No	136 (28.6%)	101 (31%)
Unknown	2 (0.4%)	0

Initial 3 ranibizumab injections, *n* (%)		
No		
1	11 (2.3%)	3 (0.8%)
2	25 (5.3%)	15 (4.1%)
Yes	435 (92.4%)	352 (95.1%)

Total number of ranibizumab injections	(≤12 months)	(12 months)
Mean ± SD	4.4 ± 1.6	4.7 ± 1.6
Range	0–10	1–10

Number of visits	(≤12 months)	(12 months)
Mean ± SD	9.4 ± 2.6	10.3 ± 1.8
Range	1–14	5–14

Discontinuation before month 12, *n* (%)	101 (21.4%)	
Reason for discontinuation, *n* (%)		
Withdrawal of informed consent	2 (0.4%)	—
Decline further injections	6 (1.2%)	—
Serious complication	1 (0.2%)	—
Retinal detachment	0	—
Referred for continuous follow up	7 (1.5%)	—
Physician's decision that no further	34 (7.2%)	—
followup is necessary		
Other reason	43 (9.1%)	—
Unknown	8 (1.7%)	—

**Table 2:** Change in VA score from baseline to last visit (on-treatment population, *n* = 370^†^).

	VA at baseline mean ± SD median (range)	VA at last visit mean ± SD median (range)	Change from baseline to last visit mean ± SD median (range)	Improved (change ≥15 letters/3 lines) *N* (%)	Stable (change <15 letters/3 lines) *N* (%)	Deterioration (change ≥15 letters/3 lines) *N* (%)
All patients	58.3 ± 12.2 60 (7–86)	59.3 ± 16.2 61 (1–90)	1.0 ± 13.6 1 (−38–56)	54 (14.7%)	273 (74.4%)	40 (10.9%)

<80 years (*N* = 195)	59.6 ± 11.1 60 (30–85)	61.6 ± 14.8 63 (16–90)	2.1 ± 12.8 3 (−37–36)	33 (16.9%)	146 (74.9%)	16 (8.2%)
≥80 years (*N* = 172)	56.8 ± 13.1 60 (7–86)	56.6 ± 17.3 59 (1–88)	−0.2 ± 14.5 0 (−38–56)	21 (12.2%)	127 (73.8%)	24 (14.0%)

Female (*N* = 245)	58.5 ± 11.6 60 (17–86)	60.3 ± 14.8 62 (1–88)	1.9 ± 12.4 3 (−38–39)	35 (14.3%)	190 (77.6%)	20 (8.2%)
Male (*N* = 122)	57.9 ± 13.2 60 (7–85)	57.2 ± 18.7 60 (5–90)	−0.7 ± 15.7 0 (−38–56)	19 (15.6%)	83 (68.0%)	20 (16.4%)

Baseline VA ≤ median (*N* = 179)	48.2 ± 8.3 50 (7–59)	51.8 ± 16.2 53 (1–83)	3.5 ± 15.2 4 (−37–56)	40 (22.3%)	120 (67.0%)	19 (10.6%)
Baseline VA > median (*N* = 188)	67.8 ± 6.0 67 (60–86)	66.4 ± 12.7 69 (23–90)	−1.4 ± 11.5 −1 (−38–21)	14 (7.4%)	153 (81.4%)	21 (11.2%)

Retrospectively recruited (*N* = 203)	58.2 ± 12.0 60 (7–86)	60.1 ± 15.6 61 (5–88)	1.9 ± 13.5 2 (−37–56)	32 (15.8%)	153 (75.4%)	18 (8.9%)
Prospectively recruited (*N* = 164)	58.4 ± 12.3 60 (21–84)	58.3 ± 17.0 61 (1–90)	−0.1 ± 13.7 0.5 (−38–33)	22 (13.4%)	120 (73.2%)	22 (13.4%)

^†^Data not available for 3 patients.

**Table 3:** VFQ-25 subscale and total scores at 3 and 12 months. Values are shown as mean ± SD.

Subscale	Month 3				Month 12			
	*n*	Baseline	Month 3	*P*-value^†^	*n*	Baseline	Month 12	*P*-value^†^
General health	130	52.5 ± 21.8	51.9 ± 20.6	0.718	89	53.9 ± 23.5	52.0 ± 23.0	0.404
General vision	129	51.0 ± 19.8	59.2 ± 18.3	<0.001	89	52.1 ± 19.7	60.2 ± 18.7	<0.001
Ocular pain	131	80.0 ± 21.8	85.0 ± 17.6	0.001	91	81.7 ± 21.1	85.7 ± 17.0	0.081
Near activities	131	58.9 ± 18.7	63.2 ± 19.0	<0.001	91	60.2 ± 18.6	64.3 ± 19.7	0.029
Distance activities	131	64.4 ± 20.3	66.7 ± 20.1	0.026	91	66.8 ± 18.8	68.1 ± 20.1	0.435
Vision specific:								
Social functioning	130	79.5 ± 21.8	80.9 ± 19.8	0.213	90	82.1 ± 19.7	82.0 ± 18.5	0.760
Mental health	131	59.6 ± 28.7	65.7 ± 27.9	<0.001	91	61.9 ± 27.3	66.0 ± 28.3	0.055
Role difficulties	129	61.7 ± 29.5	62.7 ± 29.3	0.720	89	64.9 ± 28.7	63.8 ± 28.6	0.636
Dependency	128	74.8 ± 31.7	75.6 ± 31.6	0.439	89	79.6 ± 30.5	75.8 ± 31.7	0.152
Driving	65	60.7 ± 33.8	61.1 ± 35.6	0.897	47	63.3 ± 30.2	57.6 ± 31.8	0.044
Colour vision	124	82.9 ± 19.9	83.7 ± 20.1	0.568	86	83.5 ± 18.7	81.9 ± 19.1	0.328
Peripheral vision	125	70.6 ± 20.6	72.3 ± 21.1	0.153	85	72.2 ± 19.0	72.2 ± 21.2	0.884
*VFQ-25 total*	*131*	*67.4 ± 19.3*	*70.6 ± 19.0*	*<0.001*	*91*	*69.9 ± 17.8*	*70.9 ± 18.4*	*0.389*

^†^Wilcoxon signed rank test.
